# Editorial: Current Trends in Medicinal Plant Research and Neurodegenerative Disorders

**DOI:** 10.3389/fphar.2022.922373

**Published:** 2022-06-30

**Authors:** Muhammad Ayaz, Tahir Ali, Abdul Sadiq, Farhat Ullah, Muhammad Imran Naseer

**Affiliations:** ^1^ Department of Pharmacy, Faculty of Biological Sciences, University of Malakand, Chakdara, Pakistan; ^2^ Calgary Prion Research Unit, Department of Comparative Biology and Experimental Medicine, Faculty of Veterinary Medicine, University of Calgary, Calgary, AB, Canada; ^3^ Hotchkiss Brain Institute, Cumming School of Medicine, University of Calgary, Calgary, AB, Canada; ^4^ Center of Excellence in Genomic Medicine Research, King Abdulaziz University, Jeddah, Saudi Arabia; ^5^ Department of Medical Laboratory Technology, Faculty of Applied Medical Sciences, King Abdulaziz University, Jeddah, Saudi Arabia

**Keywords:** Alzheime´s disease, natural products, neuroprotection, signaling pathways, oxidative stress

In natural product research, the ethnopharmacological approach is unique because it requires input from the cultural and social sciences. For the first time in 1967, the term “ethnopharmacology” was used as a book title “*Ethnopharmacological Search for Psychoactive Drugs”* (Efron et al., 1967). Ethnopharmacology is the scientific exploration of biologically active agents which are traditionally used or observed by man (Bruhn and Helmstedt, 1981). In many parts of the word, medicinal plants are considered as part of the traditional knowledge of a culture due to their significance in indigenous medical systems (Ayaz et al., 2019b). Thus, those studies which focus on the documentation of traditional uses of plants have ethnopharmacological relevance. The uses of medicinal plants have been described by many explorers, merchants, missionaries, and respective knowledgeable experts of healing and traditions which serve as a basis for ethnopharmacology-based drug development. Such knowledge has been widely used as a starting point for the development of drug (Heinrich, 2007).

The medicinal plants used by common people act as a significant part of all medical systems occurring in the world (Ayaz et al., 2017b; Ayaz et al., 2019c). It has been reported that in 17th century, an English housewife used Digitalis purpurea L. [Plantaginaceae] (foxglove) for the treatment of dropsy. After that, it was used by a physician WilliamWithering more systematically and he transformed this knowledge into medicine form that could be used by medical doctors (Griggs, 1981; Heinrich, 2010). Some of the ethno-pharmacologically driven natural products, identified during 19th century include morphine, emetine, strychnine, quinine, caffeine, coniine, atropine and capsaicin (Heinrich, 2010). Natural products are one of the most important sources of new drug leads. In past, crude materials isolated from various plants or their extracts were used as medicines for medical treatment and then after the second half of the 19th century due to rapid expansion of pharmaceutical industries the researchers started to develop and characterize various drugs from plant origin (Ovais et al., 2021; Heinrich, 2010). Chin et al., reported that among the marketed launched products, more than half of all new chemical entities are natural products or their derivatives (Sneader, 2005).

Since ancient times, natural products (NP) have been used as medicines to cure various illnesses (Ayaz et al., 2017a; Ayaz et al., 2020). As a source of therapeutic molecules, NP have historically proven their value and still act as an important pool for the recognition of novel drug leads (Atanasov et al., 2015). Galanthamine is a natural product obtained from several members of amaryllidaceae family and is commonly used for the treatment of Alzheimer’s disease (AD). As per the ethnobotanical information, the development of galanthamine as anti-Alzheimer’s drug consists of three main periods, including early development (for the treatment of poliomyelitis), preclinical development (as anti-Alzheimer’s drug in 1980s) and clinical development in 1990s (Heinrich and Teoh, 2004). In 1951, the acetylcholine esterase (AChE) inhibiting properties of galanthamine obtained from Galanthus woronowii Losinsk. [Amaryllidaceae] was proved by M. D. Mashkovsky and R. P. Kruglikojva-Lvov using *ex vivo* system of rat smooth muscle (Heinrich, 2010). Another example is the leaves extract of *Ginkgo biloba* L. [Ginkgoaceae], which is not considered to be a medicine in many countries but in other countries it is used to prevent dementia, memory deterioration and to enhances cognitive processes (Heinrich, 2010). Flavonoid glycosides were identified as active constituents in the leaf extracts of *G. biloba* L. in the mid of 1960 during initial research. The first patent on the complete extraction and standardization was filed in 1971 (in Germany) and 1972 (in France) (DeFeudis and Drieu, 2000). This example highlights the development of a standardized extract on the basis of traditional knowledge into an over-the-counter herbal medicine. In later years, many similar novel phytomedicines were development including *Hypericum perforatum* L. [Hypericaceae] (used for mild to moderate depression), Harpagophytumprocumbens (Burch.) DC. ex Meisn. [Pedaliaceae] (used for chronic pain), and Piper methysticum G. Forst. [Piperaceae] (used for relieving anxiety) (Collocott, 1927). Drug development for neurological disorders on the basis of ethnopharmacology persists to an exciting opportunity. According to the information available in the libraries of Swiss university, more than 150 plant species in different preparations have the potential for research and development (R&D) to develop new drugs against cognitive disorders (Adams et al., 2007).

Alzheimer’s disease (AD) is a multifactorial and progressive neurodegenerative disease. AD is the major cause of dementia and clinically characterized by loss of cognition and memory functions. Currently, there are more than 50 million AD patients affected across the globe and this number is anticipated to double every 5 years and will increase to higher than 150 million by 2050. Besides the health problem for patients and their families, AD also represents a socioeconomic burden, with estimated global costs of US$1 trillion annually, which will be doubled by 2030 (Khalil et al., 2018; Saleem et al., 2021). Neuropathologically, AD is characterized by accumulation of plaques composed of aggregated amyloid-β (Aβ) and intraneuronal neurofibrillary tangles (NFTs) of hyperphosphorylated tau proteins. In early onset familial AD, Aβ generates from the proteolytic cleavage of amyloid precursor protein (APP), by the proteolytic and enzymatic action of β- and γ-secretases, a mechanism called amyloidogenic pathway. The Aβ aggregation and deficits in Aβ clearance led to the most neurotoxic AβO species. The hyperphosphorylation of tau proteins are also associated with amyloidogenic pathway. The hyperphosphorylated tau proteins aggregate intraneuronal and forming NFTs. According to amyloidogenic pathway the elevation of AβO induces hyperphosphorylation of tau proteins, resulting intraneuronal NFTs, resulting to synaptic and neuronal degeneration and subsequently cell death (Kunkle et al., 2019; Mahnashi et al., 2021). However, more than 95% of AD cases are sporadic with late onset and very heterogeneous neuropathology. Currently, there is no cure for AD. Hence, a better understanding of the contributing factors leading to neuropathology is essential to explore the underlying causes and mediating factors to cure AD.

The purpose of this editorial is to shed light on the recent development of compounds that could prevent or treat AD. The exact underlying cause of pathological changes in AD is still unknown. However, the therapeutic strategies were applied by targeting several pathological mechanisms including protein misfolding such as aggregation of Aβ and tau proteins, pro-inflammatory mediators (IL-1β, TNF-α, TLRs, NF-kβ) and neuroinflammation, oxidative damage and accumulated reactive oxygen species (ROS) as well as its associated pathways such as heme oxygenase-1 and nuclear factor-erythroid factor 2-related factor 2 (HO-1/Nrf2), aberrant cellular and energy homeostasis signaling (e.g., AMPK, SIRT1, mTOR etc) and signalling related with elevated phosphases and kinases, including MAPK/ERK, JNK, PI3K/Akt/GSK3β, as well as synaptic trafficking and its associated pathologies (Majd and Power, 2018; Yu et al., 2021).

Aging is a process that is the reason of many diseases such as cancer, heart diseases, diabetes, and many neurological disorders such as Huntington’s disease (HD), Alzheimer’s disease (AD), and Parkinson’s disease (PD) (Tong et al., 2020). It has been reported in many studies that increased level of Reactive oxygen species (ROS) is reason of many neurodegenerative disease in different age-linked disorders such as diabetes, AD, and PD (Ovais et al., 2018; Saleem et al., 2021; Mahnashi et al., 2022). The increased ROS activate the destruction of the macromolecules such as lipids, proteins and DNA that is directly involved in the neurodegeneration through the disturbance of physiological activities of the brain (Ayaz et al., 2019a). The Research Topic, fifteen papers related to different aspects of neuroprotective drugs from natural sources were published. In the first study, Ahmad et al. reported that D-galactose (D-gal) effects neurological damage by inducing ROS signaling pathway while, Fisetin (natural flavonoid) play a protective potentials role against D-galactose-induced stress, neuroinflammation, and memory loss through adaptable anti-oxidant mechanisms, such as Sirt1/Nrf2 signaling, suppression of activated p-JNK/NF-kB signaling pathway and further downstream targets leading to inflammatory cytokines. Similarly, in another study showed neuroprotective effect of medicinal herb known as Bacopa monnieri (L.) Wettst. [Plantaginaceae], that is used as a brain tonic showed its neuroprotective effect PD when the compound extracted from Wettst extract (BME) in 1-methyl-4-phenyl-1,2,3,6-tetrahydropyridine (MPTP)-induced mice model. Further, more the BME exerts is effective and showed it neurorescue and neuroprotective and effects against MPTP-induced neurodegeneration of the nigrostriatal dopaminergic neurons. Further, it was also studied that BME help in slow down the disease progression and delay the process of neuronal damage in PD (Singh et al.). Bacopa monnieri(L.) Wettst. [Plantaginaceae] (BM) extract and the compounds isolated from it mainly used in many disease animal models. Previous studies revealed that Bacoside A may decrease the level of oxidative stress in the CNS by increasing the activities of superoxide dismutase (SOD), glutathione peroxidase (GPx), glutathione reductase (GSR) and catalase level in brain (Comens, 1983). Furthermore, BM extract was also studies in a *Caenorhabditis elegans* model of 6-hydroxydopamine (6-OHDA)-induced Parkinson’s disease (PD), and results showed that it may decrease the aggregation of α-synuclein by increasing the expression level of hsp-70 protein (Chowdhuri et al., 2002; Jadiya et al., 2011). Yet in another study, Pushparaj et al., evaluated an innovative tool (Next generation Knowledge discovery NGKD) to evaluate the AD-associated gene expression implicated in abnormal signaling pathways.

Rasool et al. have studied the role of antioxidant**s** in Schizophrenic patients. The study was carried out on 288 Schizophrenic patients of both sexes and various ages. The study reveals that there is an alteration of liver function, increase of stress marker and decrease in the level of antioxidant in the patients. It was also concluded in the study that in patients with thyroid disorder, the deficiencies of certain vitamin (B6, B9 and B12) can lead to hyperhomocysteinemia which ultimately results in the decline of antioxidants and cause oxidative disorders. Panax ginsengC.A.Mey. [Araliaceae] is a perennial plant which has wide variety of useful applications. The major components of ginseng are ginsenosides and gintonin. Li et al. has compiled a literature review on the anti-Alzheimer effect of ginseng. Their literature conclusion reveals that ginseng has therapeutic effect in neurological disorders like Alzheimer. It was further summarized that it exerts the neuroprotective effect by targeting neuro-inflammation, amyloid plaques, mitochondria and function as an antioxidant. Though there is no clinically effective drug for the management of AD. However, the summary related to the clinical findings of ginseng in the management of AD have also been compiled.

Modern society is highly advanced and has many stressful stimuli in life and these event leads to depression (Post, 1992). Mood disorders due to the stressful life are become a serious problem for health that need serious attention (Gooren and Giltay, 2014). Recently, studies in male animals model with chronic stress showed nonorganic erectile dysfunction, testicular injury, less sexual motivation was reported (Chen et al., 2019). In china, for the control of emotion and to decrease sexual dysfunction a drug name as Bupleurum falcatum L. [Apiaceae] had been widely used. Its main active component is saikosaponin D (SSD) act as antidepressant. One of the study in this Research Topic investigated that SSD exposure help to restore sexual functions after chronically stressed mice and the brain mechanisms involved in these effects (Wang et al.). Salidroside (SLDS), a phenolic glycoside compound extracted from Rhodiola rosea L. [Crassulaceae] an old medicinal plant from China has been extensively used for the treatment of multiple inflammatory diseases. Yet in another study, SLDS was showed to exhibit protective against depressive behaviors via microglia activation (Fan et al.). The study revealed that SLDS exposure significantly declined microglial immuno-reactivity for both CD68 and Iba-1. Moreover, SLDS reserved microglial activation connecting the suppression of P38 MAPK, ERK1/2, and p65 NF-κB activation and thus decreased the expression level and release of neuroinflammatory cytokines in stress mice as well as in lipopolysaccharide (LPS)-induced primary microglia (Fan et al.). Further, it was also observed that SLDS changed morphology of microglial cells by reducing the phagocytic and the decreasing the ability of attachment in LPS-induced primary microglia. The results of the study showed that SLDS exposure may improve the depressive symptoms caused by chronic stress due to the unpredictable conditions and also having the potential therapeutic application of SLDS for the treatment of depression by controlling the microglia related neuroinflammation (Fan et al.). The Catha edulis (Vahl) Endl. [Celastraceae] (Khat) is most commonly known as a stimulant. The major constituents of Khat are cathinone and cathine. Abou-Elhamd et al. have evaluated the role of Khat extract in molecular signaling using SKOV3 cells. Their observations were that the extract have significant effect on molecular level using SKOV3 cells, and thus, can cause wide variety of neurological disorders. So, in countries where Khat leaves are chewed to induce excitement and euphorbia will have severe effects on the health. Lai et al. studied effect of carnosic acid on the levodopa (L-dopa)-induced dyskinesia (LID) in rats treated with 6-hydroxydopamine (6-OHDA). They proved that by regulating the D1R signaling, CA improves the development of LID in 6-OHDA-treated rats. This leads to prevention of L-dopa-induced apoptotic cell death through modulating the ERK1/2-c-Jun and inducing the parkin. This indicates beneficial role of CA in delaying development of LID in PD patients.

Wide variety of medicinal plants with its ethnomedicinal background are a big source of drug discovery. The *Centella asiatica* (L.) Urb. [Apiaceae] have been explored to have neuroprotective and anti-inflammatory properties. The plant exert its effect by protecting the mitochondria and have antioxidant properties (Wong et al.). Lee et al. tested herbal extract from *Glycyrrhiza* uralensis Fisch. ex DC. [Fabaceae], Atractylodes macrocephala Koidz. [Asteraceae], Panax ginseng C.A.Mey. [Araliaceae], *Astragalus* mongholicus Bunge [Fabaceae] to study the anti-inflammatory in the Muscle and Spinal Cord of an Amyotrophic Lateral Sclerosis Animal Model. They performed behavioral tests, including rotarod test and foot printing, immunohistochemistry, and Western blotting, in hSOD1^G93A^ mice. Their experiments resulted in improved motor activity and reduced motor neuron loss in hSOD1^G93A^ mice. They also found that the herbal extract reduced levels of oxidative stress-related proteins (HO1, NQO1, Bax, and ferritin) and inflammatory proteins ((GFAP, CD11b, and TNF-α)) in the skeletal muscles and spinal cord of hSOD1^G93A^ mice.

Cerebral amyloid angiopathy (CAA) is considered by the accretion of β-amyloid (Aβ) in the walls of cerebral vessels, further causing the complications such as convexity subarachnoid hemorrhage, intracerebral hemorrhage as well as cerebral microinfarcts (Love et al., 2014). Dementia and strokes may develop in the patients with CAA-related intracerebral hemorrhage. Many experimental studies explained and demonstrated the pathology of more than 90% of AD patients have associated with CAA and leading to common pathogenic mechanisms. Possible causes of CAA include impaired Aβ removal from the brain through the system called as intramural periarterial drainage (IPAD) (Saito et al., 2019). Moreover, CAA causes control of IPAD causing the limiting clearance. Early interference in CAA may help in the prevention of AD. In another paper published in this Research Topic, Saito et al., summarized that Taxifolin (dihydroquercetin) is a plant flavonoid is a safe and effective therapy for CAA. Taxifolin is a flavonoid extracted from plant is widely existing in the supplement product, which has been used to exhibit against anti-inflammatory effects, anti-oxidative effect and used as protective agents against the advanced glycation end products as well as mitochondrial damage. Further the flavonoid also showed that it help to facilitate disassembly and prevent oligomer formation and increase clearance of Aβ in CAA of mouse model. Taxifolin treatment also prevent the spatial reference memory impairment and cerebrovascular reactivity in CAA animal model. Further studied required to prove and explain the exact mechanism of Taxifolin that will help to use this drug with effectiveness and safe for the patients with CAA Saito et al. Corona virus disease (COVID-19) is a pandemic of the current era. The COVID-19 has the symptoms from simple common cold to more complex and even leading to the neuro-COVID complications. Pushparaj et al. has worked on the gene sequencing targeting the neuro-COVID. They were able to embark RNA sequencing and find out that some small organic molecules from natural or synthetic source can be useful in the treatment of neurological disorders related to COVID-19. Neuroprotective and anti-inflammatory effect of Pterostilbene was tested against Cerebral Ischemia/Reperfusion injury via suppression of COX-2 in middle cerebral artery occlusion (MCAO) rodent model by Yan et al. Treatment of Pterostilbene significantly reduced neurological score, infarct volume and brain edema. Hepatic parameters (ALT, AST and ALP), renal parameters (uric acid, creatinine, BUN and urea), lipid parameters (TG, HDL, LDL, TC and VLDL), antioxidant parameters (SOD, CAT, GSH, GPx, MDA), inflammatory cytokines (TNF-α, IL-1β, IL-6, and IL-10), inflammatory mediators (COX-2, PGE_2_, iNOS) AND metalloproteinases (MMP) (MMP-2, and MMP-9) levels were improved. Results of these studies show that Pterostilbene is effective in the treatment of cerebral ischemic stroke and cerebral ischemia reperfusion.

Cerebral hypoperfusion (CH) causes neurological diseases like Alzheimer’s-type dementia and vascular cognitive impairment and dementia. To find plant-based treatment for this problem, Liu et al. carried out experiments to unearth potential of Cucurbitacin E (steroidal tetracyclic terpene) in a rat model of CH. Treatment of the rats with Cucurbitacin E (CuE) for 28 days resulted in reduced CH-Induced neurological, sensorimotor and memory deficits, low lipid peroxidation (TBARS content) and protein carbonyls, increased GSH and catalase and diminished inflammatory cytokines (TNF-α, NF-κB, MPO, MMP-9, and iNOS). LDH, caspase-3, glutamate and acetylcholinesterase activities were decreased in Cu-E treated rats subjected to CH. Viable neuron density in the cortex was increased after treatment with CuE. These findings suggest that CuE is a potential compound against CH-associated disorders.
